# Daily Exceedance of World Health Organization Air Pollutant Guidelines and Cognitive Aging

**DOI:** 10.64898/2026.09.23.26363787

**Published:** 2026-09-29

**Authors:** Mikaela Bloomberg, Giorgio Di Gessa, Shaun Sholes, Antonio Gasparrini, Arturo de la Cruz Libardi, Andrew Steptoe, Paola Zaninotto

## Abstract

**Importance:**

Long-term exposure to higher average air pollutant concentrations is associated with poorer cognitive health, but annual averages can obscure substantial differences in day-to-day exposure. Whether recurrent high pollution days are associated with long-term cognitive aging trajectories among people with similar overall air pollution exposure is unclear.

**Objective:**

To determine whether the annual number of days exceeding World Health Organization daily guideline values for nitrogen dioxide (NO _2_ ) and particulate matter (PM _2.5_ and PM _10_ ) is associated with subsequent cognitive decline after accounting for annual mean air pollutant exposure.

**Design:**

Prospective cohort study using data from waves 1-10 (2002-03 to 2021-23) of the English Longitudinal Study of Ageing.

**Setting:**

Population-based cohort of community-dwelling adults in England.

**Participants:**

12,235 adults aged 50+ years at analytic baseline.

**Exposures:**

Number of days exceeding World Health Organization daily guideline values (‘exceedance days’) for NO _2_ , PM _2.5_ , and PM _10_ , derived from residence-linked environmental estimates during the calendar year preceding the analytic baseline.

**Main Outcomes and Measures:**

Baseline cognitive performance and model-estimated 14- year cognitive decline (corresponding to the 75th percentile of follow-up duration) in episodic memory and verbal fluency.

**Results:**

Exceedance days were not associated with baseline memory or verbal fluency after adjustment for annual mean air pollutant exposure. More NO _2_ exceedance days were associated with faster cognitive decline: comparing the 75th percentile with the median of NO _2_ exceedance days, the difference in 14-year decline was -0.09 SD (95% CI, -0.17 to -0.01; p = 0.02) for memory and -0.10 SD (95% CI, -0.19 to 0.00; p = 0.04) for fluency. PM _2.5_ and PM _10_ exceedance days were not associated with cognitive decline.

**Conclusions and Relevance:**

In this cohort study of older adults, a higher number of NO _2_ exceedance days was associated with faster subsequent cognitive decline after adjustment for annual mean air pollutant exposure. This suggests that daily exposure to high NO _2_ concentrations may be related to accelerated cognitive aging even when offset by lower exposure on other days, potentially providing a new rationale for identifying and prioritizing areas with recurrent high NO _2_ exposure and the local emission sources contributing to it.

**Key points:**

**Question**

Among individuals with similar average air pollution exposure but different day-to-day exposure patterns, are more high pollution days associated with faster cognitive decline?

**Findings**

In this UK-based cohort of 12,235 adults aged 50+, individuals exposed to more days on which nitrogen dioxide (NO _2_ ) concentrations exceeded World Health Organization guideline values subsequently had significantly faster cognitive decline over up to 18 years, after adjustment for annual mean air pollutant exposure. No associations were observed for particulate matter.

**Meaning**

Daily exposure to high NO _2_ concentrations is associated with faster cognitive decline even when offset by lower concentrations on other days.

## Full Text Availability

The license terms selected by the author(s) for this preprint version do not permit archiving in PMC. The full text is available from the preprint server.

